# Temperature and light effects on *Trichobilharzia szidati* cercariae with implications for a risk analysis

**DOI:** 10.1186/s13028-020-00553-z

**Published:** 2020-09-15

**Authors:** Azmi Al-Jubury, Per Kania, Anette Bygum, Kurt Buchmann

**Affiliations:** 1 Laboratory of Aquatic Pathobiology, Department of Veterinary and Animal Sciences, Faculty of Health and Medical Sciences, University of Copenhagen, Stigbøjlen 7, 1870 Frederiksberg C, Denmark; 2 Department of Dermatology and Allergy Centre, Odense University Hospital, J.B. Winsløws Vej 4, 5000 Odense C, Denmark

**Keywords:** *Trichobilharzia*, Bird schistosomes, *Lymnaea stagnalis*, Cercaria, Swimmer’s itch

## Abstract

**Background:**

Cercarial dermatitis (swimmer’s itch) caused by bird schistosome cercariae, released from intermediate host snails, is a common disorder also at higher latitudes. Several cases were observed in the artificial Danish freshwater Ringen Lake frequently used by the public for recreational purposes. The lake may serve as a model system when establishing a risk analysis for this zoonotic disease. In order to explain high risk periods we determined infection levels of intermediate host snails from early spring to late summer (March, June and August) and elucidated the effect of temperature and light on parasite shedding, behavior and life span.

**Results:**

Field studies revealed no shedding snails in March and June but in late summer the prevalence of *Trichobilharzia szidati* infection (in a sample of 226 pulmonate *Lymnaea stagnalis* snails) reached 10%. When investigated under laboratory conditions the cercarial shedding rate (number of cercariae shed per snail per day) was positively correlated to temperature raising from a mean of 3000 (SD 4000) at 7 °C to a mean of 44,000 (SD 30,000) at 27 °C). The cercarial life span was inversely correlated to temperature but the parasites remained active for up to 60 h at 20 °C indicating accumulation of cercariae in the lake during summer periods. Cercariae exhibited positive phototaxy suggesting a higher pathogen concentration in surface water of the lake during daytime when the public visits the lake.

**Conclusion:**

The only causative agent of cercarial dermatitis in Ringen Lake detected was *T. szidati.* The infection risk associated with aquatic activities is low during spring and early summer (March-June). In late summer the risk of infection is high since the release, behavior and life span of the infective parasite larvae have optimal conditions*.*

## Background

A series of schistosome species within the genus *Trichobilharzia* use birds (water fowl) as final hosts, carrying the adult stage, and pulmonate snails as intermediate hosts, shedding infective cercariae. These parasites are zoonotic as human may act as accidental host. The cercariae are the causative agent of cercarial dermatitis (swimmer's itch), a skin rash provoked by penetration of cercariae into the skin. Cercarial dermatitis has been recorded worldwide [1–4] including cold areas at northern latitudes e.g. Denmark [5–7], Norway [8], Sweden [9] and Iceland (geothermally heated water bodies) [2]. Cercarial dermatitis is diagnosed mainly during summer time when both cercarial shedding from snails and recreational activities (bathing, swimming, fishing, sailing) in freshwater bodies are peaking [3, 10–12]. During the warm summer of 2018, several clinical cases were reported among children and adults following bathing in Ringen Lake, Roskilde, Denmark [3]. The lake is frequently visited by residents in the area for swimming and fishing, and may serve as a model system for establishing a risk analysis. The bird schistosomes within the genus *Trichobilharzia* spp. reside as adult egg- laying parasites in internal organs (veins of viscera or the nasal mucosa) of the definitive bird host. The eggs release ciliated larvae (miracidia) which are released to the water body and subsequently penetrate the intermediate snail host whereafter asexual development in sporocysts lead to production of cercariae. These are shed into the water ready to penetrate the skin of the definitive host for completion of the life cycle [1]. However, only a few of the cercariae reaches its final or accidental host, because cercariae are directly exposed to and affected by environmental biotic and abiotic factors which may affect survival and life span [13, 14]. We have therefore conducted an analysis of the bird schistomes in the intermediate host snails sampled in the lake from early spring to late summer (March, June and August). As the cercarial dermatitis cases were reported in the summer period [3] it is relevant to elucidate the behaviour of the cercariae in relation to temperature (effect of different temperatures on cercarial shedding and life span) and light (phototaxy). Based on the results we present a risk analysis with recommendations for management of such a lake system and instructions for the public in order to prevent or reduce occurrence of future cercarial dermatitis cases.

## Methods

### Location

Ringen Lake is an artificial freshwater lake (supplied with ground water) located in Roskilde, Denmark (55°37′56.3"N 12°04′55.0"E). It has a short history, as it was established between 1973 and 1977. It has a surface area just below 10,000 m^2^, a maximum water depth of 1.5 m and is used for leisure and recreational purposes. Several species of aquatic plants and animals including invertebrates (snails), fish (roach, perch and pike) and water fowl (ducks and swans) have populated the lake. The water temperature during snail collections in August 2019 was 18–19 °C at various positions in the lake (measured at a depth of 1 m) and the photoperiod 15 h light and 9 h darkness.

### Collection of snails

Pulmonate snails (mean shell length and width 44.6 ± 6.3 and 22.2 ± 3.6 mm, respectively) were randomly collected by hand along several parts of the shore in March, June and August of 2019. The samples size in March was low due to the reduced availability of snails in that month (probably associated with the low water temperature at 7 °C), while snails were abundant at higher temperatures later in the season (Table 1).

**Table 1:** Pulmonate snails collected at different time of the year in Ringen Lake with prevalence % of *Trichobilharzia* and water temperature at time of collection

Time/month	collected snails	*Trichobilharzia* spp. infected snails	Non-infected snails	Prevalence%	Water temperature (°C)
March	42	0	42	0	7
June	91	0	91	0	13–16
August	226	22	204	10	18–19
Total	359	22	337	6	

### Identification of snails

After transportation to the Laboratory of Aquatic Pathobiology at the University of Copenhagen, infected snails were identified based on morphometric characters (morphology, shell length and width) [15]. Snails were screened for release of *Trichobilharzia* sp. cercariae by placing them separately in 400 mL plastic beakers containing 100 mL dechlorinated water at 20 °C (room temperature) exposed to daylight for 6 h. Subsequently, water was examined for presence of released cercariae under a dissecting microscope (Leica CLS 150X, magnification ×4–40).

### Identification of cercariae

**Fig. 1:**
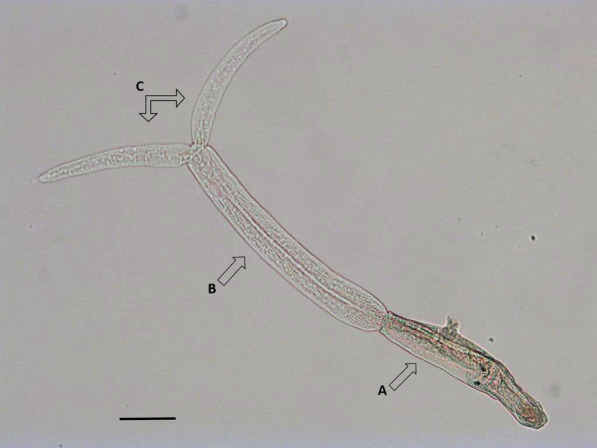
*Trichobilharzia szidati* released from *Lymnaea stagnalis* snail at Ringen Lake, Roskilde, Denmark. **a** Frontal part of the cercaria with the part penetrating host skin, the ventral sucker for attachment of the cercaria pre-penetration and two eye-spots. **b** Tail. **c** Furca. Scale bar: 100 µm

The released cercariae were identified to type and genus level based on standard morphometric characters [1, 16–18]. In brief: Cercariae released from each snail were conserved in 70% ethanol and subsequently mounted on slides in glycerine-jelly (Fig. 1). Measurements (µm) (forebody length and width, tail stem length and width, furca length, distance from apical end to ventral sucker) were done by use of a light microscope (Olympus, CH30PF200).

Species identification was based on PCR and sequencing of the gene locus ITS rDNA performed according to [7]. In brief: DNA was extracted from individual cercariae, isolated from each of 12 individual infected snails used in the experiment, using QIAamp®DNA Mini Kit from (QIAGEN). PCR was performed using the forward primer D1 (5′AGGAATTCCTGGTAAGTG CAAG′3) and the reverse primer BD2 (5′TATGCTTAAATT CAGCGGGT′3) [19]. Reactions were run in a T100™ Thermal Cycler (BioRad, Denmark) in a total reaction volume of 60 μL containing: 1 μM of each primer (Sigma-Aldrich, Denmark), 1 mM of dNTP-mix (Life Technologies, Denmark), 1.25 units of BIOTAQ DNA polymerase (DNA Technology A/S, Denmark), 1.5 mM MgCl_2_, and 6 μL 10 × PCR buffer and 2 μL DNA template was added. Finally, DNase and RNase free water (Invitrogen™, Life Technologies, Denmark) was added to a final reaction volume of 60 μL. Sterile water was used as negative control. The following PCR protocol was used: initial denaturation at 94 °C for 5 min, followed by 45 cycles of denaturation at 94 °C for 30 s, annealing at 57 °C for 30 s, and elongation at 72 °C for 75 s. After cycling, a final elongation step at 72 °C for 7 min was performed. The PCR products were visualized by gel electrophoresis and subsequently purified using Illustra™ GFX™PCR and Gel band purification kit (VWR, Denmark). Fragments were sequenced by Macrogen Inc. (Seoul, South Korea) using the PCR primers used for the amplification process. Complete sequences were submitted to GenBank.

### Shedding experiments

*Trichobilharzia* sp. infected snails were isolated in individual shedding beakers (diameter 55 mm, depth 70 mm, maximum water capacity 166 mL) containing 100 mL dechlorinated water and used for determination of temperature and light effects on cercarial shedding. Snails were fed lettuce (*Lactuca sativa*) during the experiments.

### Enumeration of cercariae

The number of cercariae released from each individual snail was quantified by daily exchange of incubation water (by moving the snail to a new plastic beaker contain similar water temperature). Three subsamples, each of 100 µL, from each snail beaker were taken with a pipette after stirring the water with released cercariae. Formaldehyde (4%) were added to the subsamples to kill and fix the cercariae whereafter they were enumerated under the dissection microscope (Leica CLS 150 X). The total number of cercariae released per day was then calculated.

### Influence of temperature on cercarial shedding from snails

Ten snails, each in its own beaker, were investigated at each temperature starting with 7 °C and after 24 h shedding the snails were incubated at 10 °C and shedding was observed for 24 h and so forth. So the procedure was conducted with ten snails at five different temperatures: 7, 10, 15, 22, 27 °C under a 15 h light: 9 h dark cycle (in thermostat controlled rooms). The cold light source was Cool white Philips LEDtube light 1200 mm UO 16 W 840 T8. Every 24 h water was exchanged (by moving the snail to a new plastic beaker with a similar water temperature) whereafter the exchanged water was examined under the dissecting microscope for enumeration of released cercariae. Experiments were conducted over five consecutive days.

### Influence of light on cercarial shedding from snails

#### Light–dark cycle 12:12 h

Four infected snails were incubated in individual shedding beakers at room temperature (20 °C) applying a 12 h light:12 h dark cycle for 7 days (natural daylight regime). The number of released cercariae per snail per 12 h (each light and dark period) was noted.

#### Constant light- dark and dark–light cycle 3:3 days

Six infected snails were divided into two groups both kept at 20 °C. Group one: each of three snails were incubated in individual shedding beakers placed under constant cold light (Cool white Philips LEDtube light 1200 mm UO 16 W 840 T8). Group two: each of three snails were kept under corresponding conditions but in constant darkness. Both groups were kept for 3 days whereafter illumination was reversed for three days (group one was placed in dark and group two in light). The number of released cercariae per snail per 24 h was noted.

### Phototaxy

**Fig. 2:**
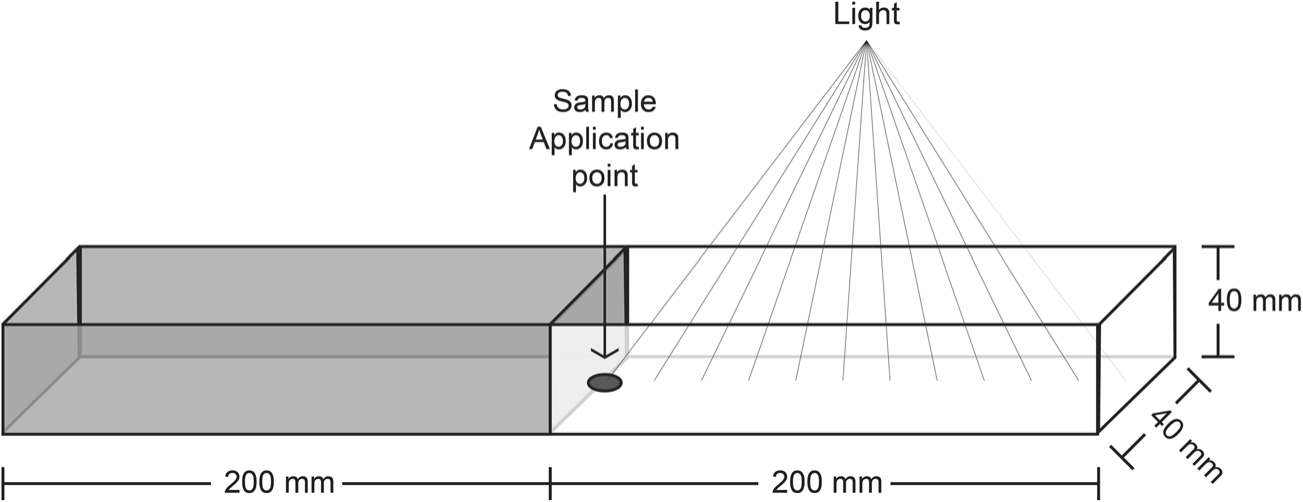
Phototaxy plastic chamber used to determine effects of light on cercarial behavior, comprising two zones each with a length of 20 cm. One zone was dark (covered with black plastic) and the other was uncovered and exposed to light. Illumination (cold light from a Leica microscope) was placed in the light end. Cercariae were placed by pipette on the border between zones (dark spot). The location of 60 cercariae was determined after 60 min incubation

The experiment was conducted at 20 °C in a plastic chamber (dechlorinated water level 2 mm) designed for this study. Dimensions were 40 cm length × 4 cm width × 4 cm height (wall thickness 2 mm) (Fig. 2). The chamber was divided equally into two 20 cm zones. One was with black covering (dark zone), while the other zone was kept uncovered and illuminated (Fiber Optic Cold Light Source Kaltlichtquelle 150 W). The chamber was placed and leveled under a Leica CLS 150X dissecting microscope for location and enumeration of cercariae. For each trial a total of 50 cercariae (1–6 h post-shedding in daylight at 20 °C) were applied by pipette directly into the borderline between the light and dark zones. The location and number of cercariae (presence in dark or light zone) were then recorded under the microscope after 60 min incubation.

### Life span of cercariae

The life span of cercariae shed from infected snails at 20 °C was examined at 5, 10, 15, 20, 25 and 30 °C. A total of 18 shedding beakers, each containing 50–60 cercariae (1–6 h post-shedding) in a volume of 30 mL dechlorinated tap water, was used. Three beakers were kept and examined for living cercariae every 12 h at each of the specified temperatures (thermostat controlled rooms). Cool white Philips LED tube light 1200 mm UO 16W 840 T8) was used for illumination. Immobility despite mechanical stimulation (touch with a needle) was used as criterion for death. Inactive cercariae were tested and recorded alive if they could be stimulated to move.

### Statistics

We used a linear regression and Pearson’s correlation test to investigate relationship between daily cercarial release at different temperatures. Differential cercarial shedding from infected snails kept at dark and light conditions were tested by use of Student’s t-test. Likewise, location of cercariae in dark or light zones after 60 min incubation was compared by a Student’s t-test. Differential survival according to temperature was evaluated using ordinary one-way ANOVA. Point-to-point lines are presented whereby time points with 10% cercarial survival can be determined (Fig. 7). These time points were plotted against temperature and linear regression performed (Fig. 7 insert). All the statistical analyses were performed using Graph Pad Prism version 7 and a 5% probability level was used in all analyses.

## Results

### Identification of snails

All pulmonate snails collected in Ringen Lake were identified as *Lymnaea stagnalis* based on the characteristic features of the snail shell [15]. Their shell length and width (mean ± SD) were 44.6 ± 6.3 and 22.2 ± 3.6 mm, respectively. A total of 20 snails were used for further laboratory studies on temperature and light dependent shedding.

### Identification of cercariae

All cercariae belonging to the genus *Trichobilharzia* shed from the investigated *L. stagnalis* were identified as *Trichobilharzia szidati*. Details of morphometric characters are provided in Table 2.

**Table 2:** Morphometric characters of cercariae released by *L. stagnalis* collected in Ringen Lake

	Mean ± SD
Total length	952 ± 86
Body length	321 ± 44
Body width	54 ± 5
Tail stem length	386 ± 49
Tail stem width	40 ± 8
Furca length	254 ± 19
Distance from apical	225 ± 33
body end to ventral sucker	

Dimensions of *Trichobilharzia szidati* cercariae. Mean and SD of a total of 12 specimens

BLAST analyses were conducted with recovered sequences of the complete ITS1, 5.8S and ITS2 rDNA region at GenBank (NCBI). In all cases, a high identity (> 99%) was found with *T. szidati* GenBank accession no. GU233735 (Czech Republic).

### Influence of temperature on *Trichobilharzia szidati* cercarial shedding

**Fig. 3:**
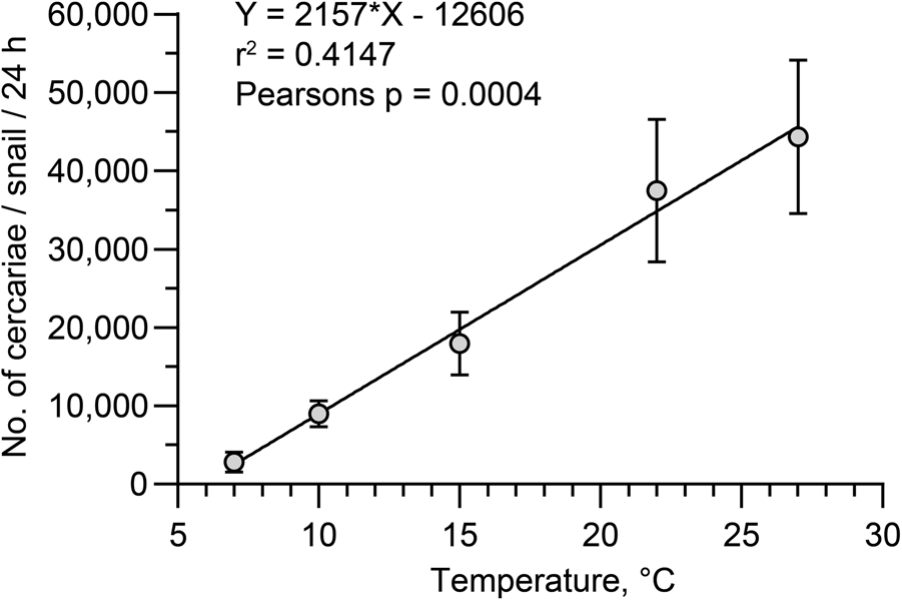
Number of released cercariae of *Trichobilharzia szidati* per infected snail per day at 7, 10, 15, 22, 27 °C. Each point represents mean and SD of three snails (r^2^: Pearson’s coefficient of correlation)

Cercarial emission from *Lymnaea stagnalis* snails was positively correlated to temperature. The daily shedding reached a mean of 3000 (SD 4000) cercariae per snail per day (minimum 20) at 7 °C and a mean of 44,000 (SD 30,000) with a maximum of 77,000 cercariae per snail per day at 27 °C. Accordingly, the mean numbers of cercariae released from one specimen of *L. stagnalis* daily were 37,000, 18,000, and 9000 at 22, 15, 10 and 7 °C, respectively (Fig. 3).

### Influence of light on cercarial shedding from *L. stagnalis*

**Fig. 4:**
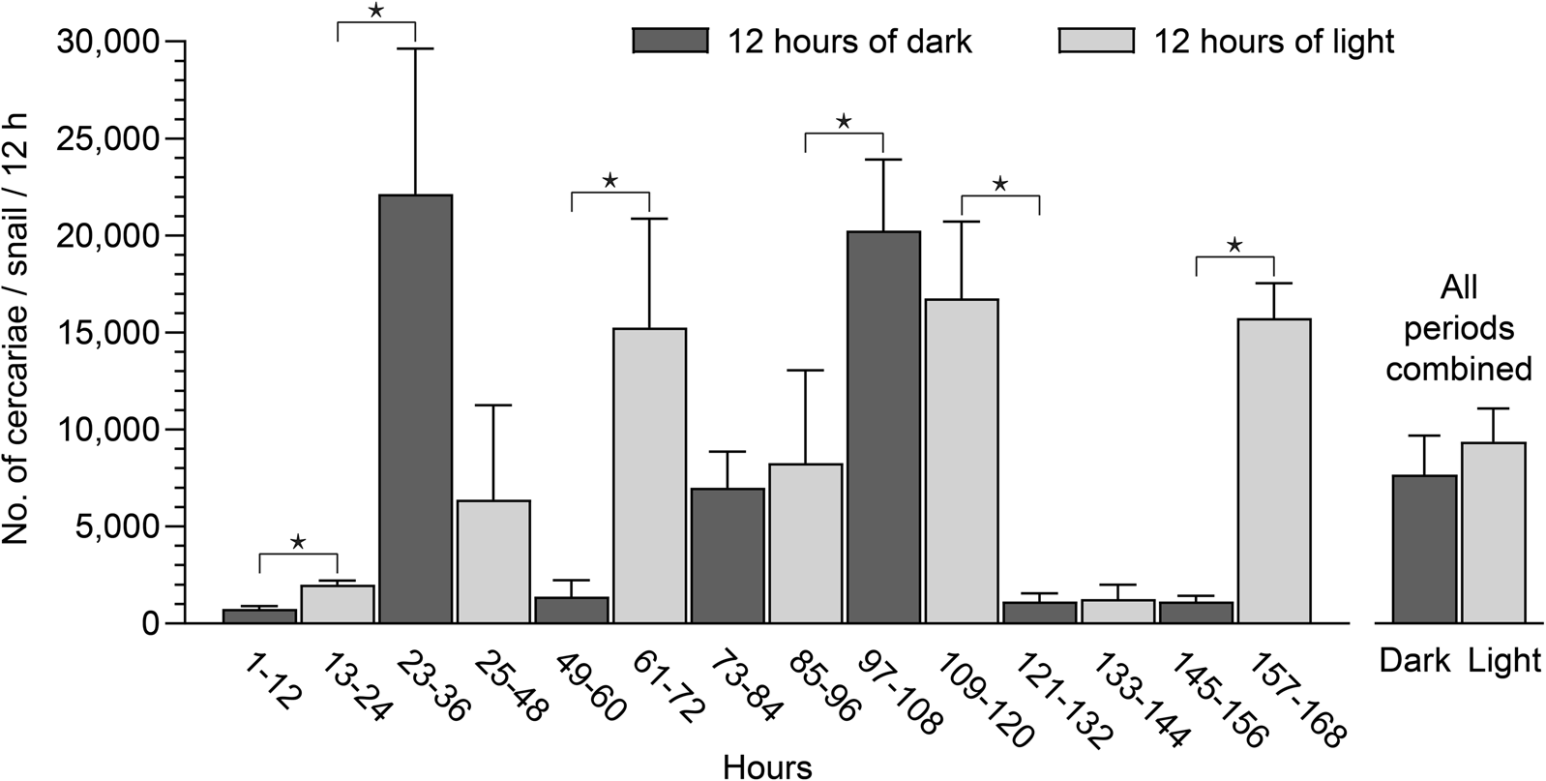
Shedding of *Trichobilharzia szidati* cercariae from *Lymnaea stagnalis* snails in 12 h light and 12 h dark periods for 7 days. Each column represents mean and SD of three snails. Insets right: Average of 14 counts. *: P ˂ 0.05

**Fig. 5:**
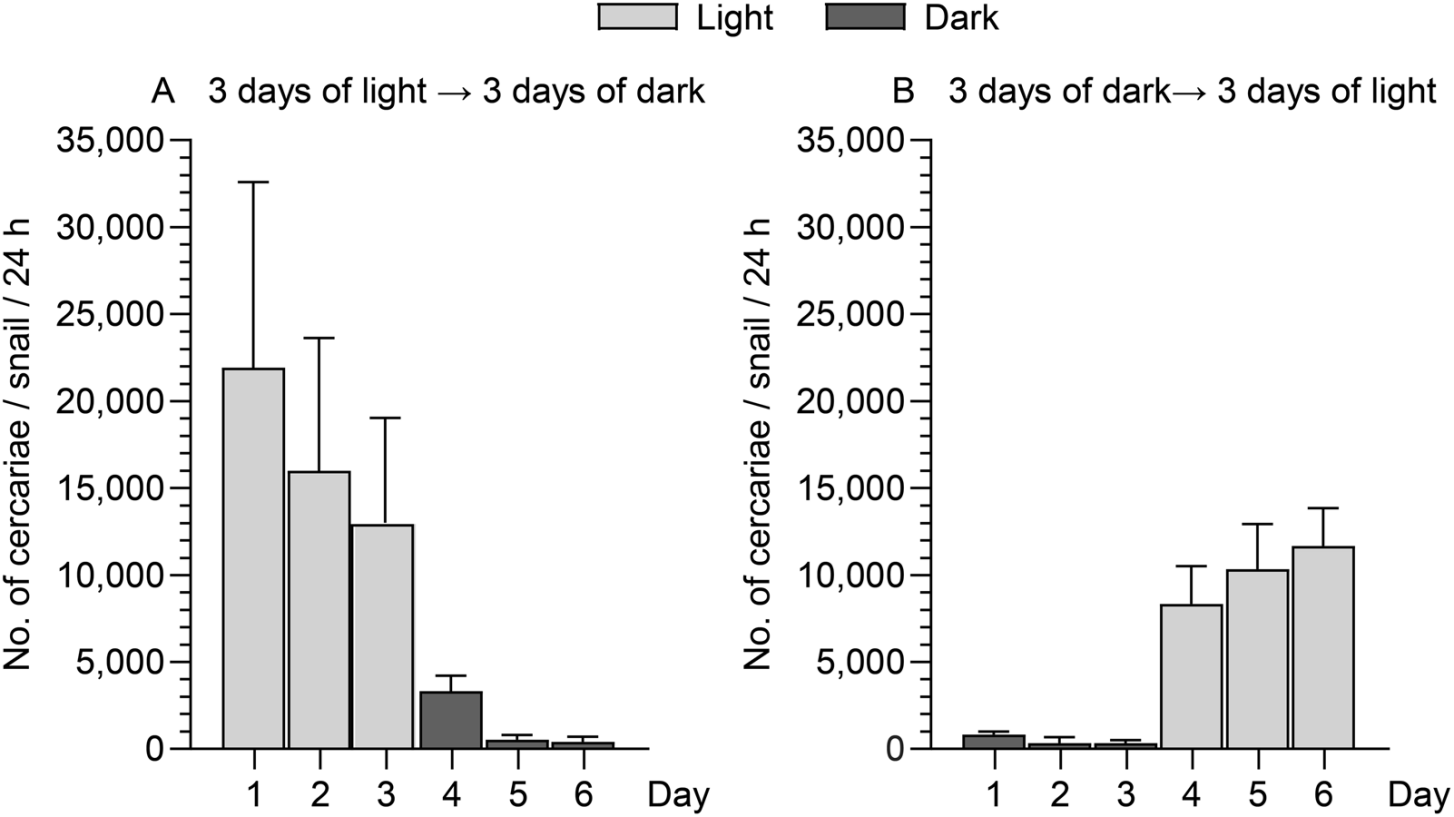
Shedding of *Trichobilharzia szidati* cercariae per 24 h during six days. Each column represents mean and SD of shedding from six snails per day at 20 °C. **a** 3 days constant light or darkness, **b** 3 days constant darkness or light

The cercarial shedding from *L. stagnalis* was examined in shifting light and dark periods (light dark cycle of 12:12 h) during 7 days in the laboratory at 20 °C but no clear release pattern was observed (Fig. 4). In contrast, when infected snails were kept constantly for 3 days, either in light or dark conditions, it was indicated that darkness inhibited shedding and light stimulated shedding under these circumstances (Fig. 5).

### Phototaxy

**Fig. 6:**
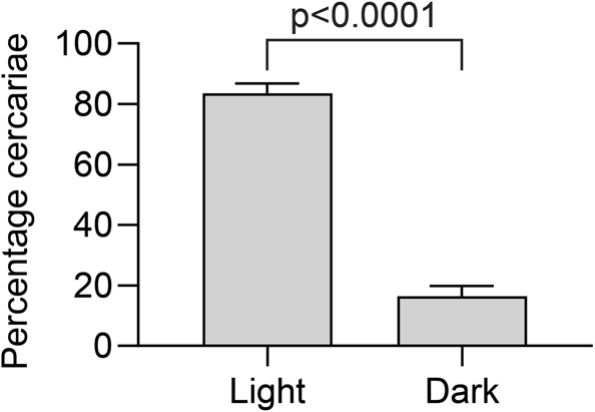
Percentage of *Trichobilharzia szidati* cercaria in light and dark zones after 60 min incubation. Each column represents mean and SD of 20 measurements

*Trichobilharzia szidati* cercariae were attracted to light as significantly more cercariae (83%) were recorded in the light zone as compared to the dark zone (17%) (Fig. 6).

### Life span of *Trichobilharzia szidati* cercariae

**Fig. 7:**
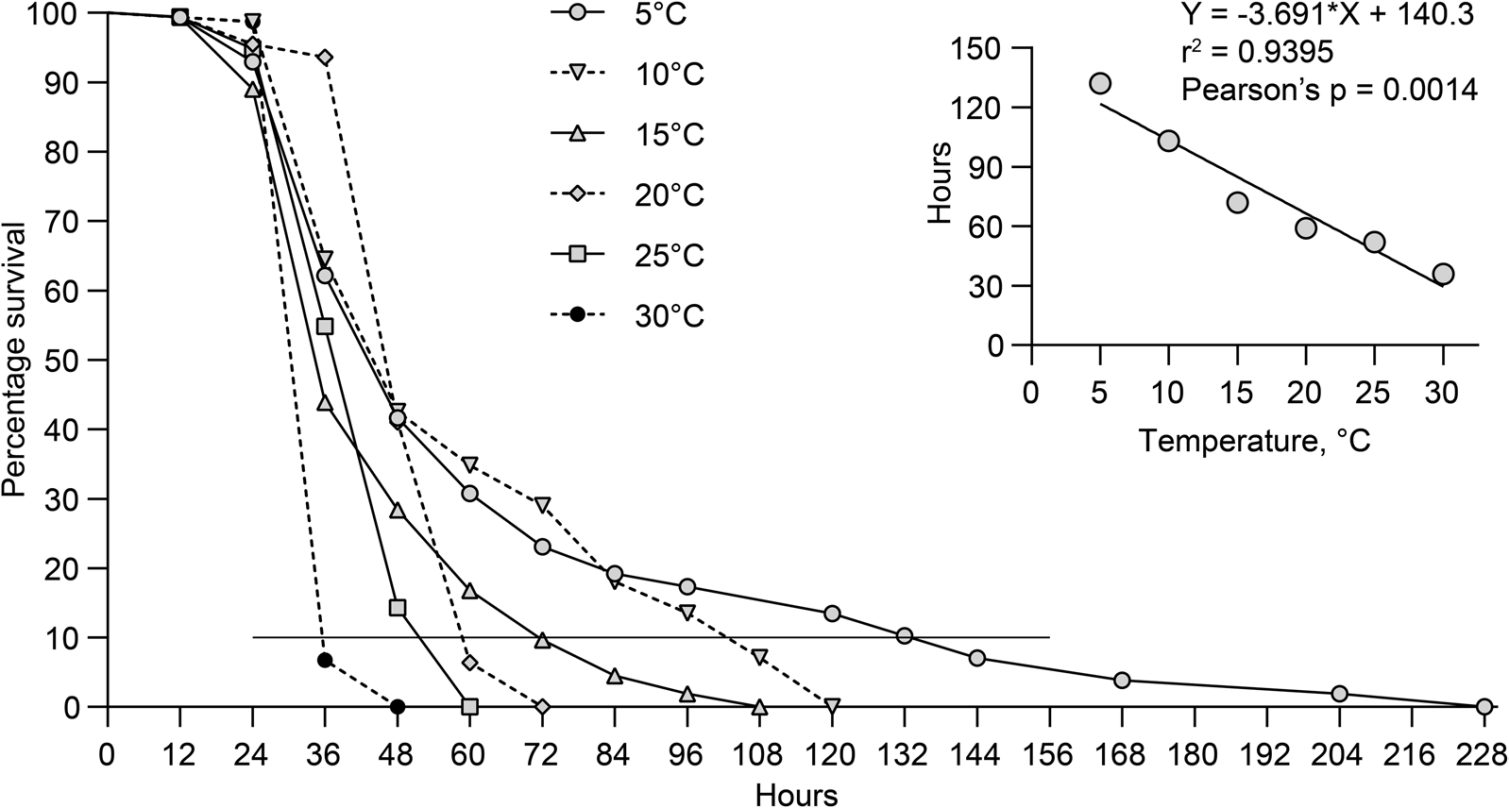
Influence of temperature on the survival of *Trichobilharzia szidati* cercariae at 5 different temperatures during a 228 h period. Each point represent percentage of living cercariae out of 60 (percentage survival). Insert show correlation between survival time (h) and temperature. r^2^: Pearson’s correlation coefficient

The cercarial life span was negatively correlated to temperature as the longevity decreased with increasing water temperature. The maximum life span recorded was 204, 108, 96, 60, 48, and 36 h at 5, 10, 15, 20, 25 and 30 °C, respectively (Fig. 7).

## Discussion

The investigated Ringen Lake is an artificial lake which was established in 1977 for recreational purposes including bathing and other aquatic activities. The public (children, adults, bathers, swimmers, anglers) is attracted to the shallow lake especially during warm summer periods. In the warm summer of 2018, cases of cercarial dermatitis (swimmer’s itch) following contact with the lake were reported [3]. In order to describe the causes, provide a risk analysis and develop a preventive control strategy we investigated the occurrence of intermediate snail hosts, the identity of the causative pathogen and determined effects of temperature and light on shedding of infective cercariae from the host snails. This will provide a basis for evaluation of the infection risk during different seasons and during the day. The pulmonate snail *L. stagnalis* was the only snail species observed in the lake, and it is worthwhile to emphasize the uniqueness of the observation of only one species of *Trichobilharzia* in the investigated lake. Several species within the genus have previously been recorded in other natural Danish lakes [6, 7]. It may be explained by the generally low biodiversity in the lake due to its short history and its artificial nature. When performing shedding experiments using these snails the only isolated parasite species, with a potential to elicit cercarial dermatitis, was *T. szidati*. A series of previous investigations have indicated that the release of infective cercariae and their behavior is dependent on temperature and/or light conditions [20, 21] and we therefore conducted laboratory studies on how could these ecological factors may influence the occurrence of infective cercariae in the lake. Field samplings from Ringen Lake, comprising several hundreds of snails, showed a seasonal occurrence of cercaria releasing snails, since only samplings in August—but not in March and June—were positive. It cannot be excluded that snails collected in March and June carried immature developing sporocysts (stage producing cercariae) [22] but in August the prevalence of shedding snails was 10%, confirming presence of fully mature sporocysts. Temperature may affect development of all parasitic stages of the life cycle [23–25] whereby cercariae may occur earlier in warm summers. Under all circumstances, when sporocysts are mature, as in August 2019, we found that the shedding rate was correlated with temperature. The highest mean cercarial shedding (44,000 (SD 30,000) cercariae per day per snail), was recorded at 27 °C and the lowest mean rate (3000 (SD 4000) cercariae per snail per day) at 7 °C. This temperature dependence is a general observation in trematode infected snails [20, 21] and explain why the infection risk is higher during warm periods when the public seek the lake. When evaluating infection risks the life span results indicate that cercariae survive several days after emergence from the snail. Even at high summer temperatures (25 and 30 °C) a part of the cercariae stay alive for both 48 h and 36 h, respectively, and at lower temperatures they survive for more than eight days. Further, light is an abiotic factor with a possible effect on the infection level in lakes. We assessed the effect of the light-darkness cycle on release of cercariae and thereby potentially the infection risk. Diurnal shedding variations have been suggested as a synchronization of cercarial shedding with the daily activity patterns of the definitive host. Thus, ducks acting as the natural final host of bird schistosomes show activity patterns around sunrise and sunset [26] and synchronization of cercarial shedding with host activities can be explained as an adaptive behavior which can increase the probability of transmission [27]. Several examples are known from other schistosome parasite-host systems where some species emerge from snails at night (*S.douthitti*), midday (*S. mansoni*), later in the afternoon (*S. japonicum*), and some throughout the day (*T. physellae*) as cercarial release is related to behavioral patterns of the specific definitive host [28]. Emergence of *T. szidati* in German water bodies was also reported to occur during the first hours of light exposure [29]. In the present study *T. szidati* release was shown to be independent of light-darkness cycles unless the light or dark periods were of several days corresponding to previous other studies [30]. The inhibition of shedding in the laboratory setting with an extended dark period and stimulation of shedding in the light may indicate that light has an effect, although it has no importance for the infection risk in the Ringen Lake area. The behavior of the free-living cercarial stage may play a key role in the parasite transmission [31] but schistosomes differ with respect to light responses, with some species being less sensitive to light [32]. We noticed a positive phototactic behavioural pattern of *T. szidati* suggesting that the cercariae possess photoreceptors corresponding to previous findings in *Trichobilharzia physellae* [33]. *Trichobilharzia ocellata* was reported to respond to light with complex behaviors and not always showing phototropism [34]. Positive phototaxy may be considered part of a life cycle strategy increasing the probability to find a host and complete the life cycle. In the investigated Ringen Lake it may have implications for the risk analysis that cercariae concentrate in upper water layers during daytime when bathers visit the lake.

The longevity of *T. szidati* cercariae is temperature dependent as cercariae kept at 5 °C survived more than eight days while they died after 48 h at 30 °C. This progressive decrease in the life span of the free-living cercariae corresponds to findings in other trematode species. The negative correlation between lifespan and temperature is probably due to the temperature dependent usage of the stored energy reserves (glycogen) in the parasite [20, 21, 35]. Ringen Lake can serve as a suitable model system for establishing risk analysis strategies with regard to cercarial dermatitis. When providing an overall assessment of the risk associated with aquatic activities (bathing, swimming, fishing) in Ringen Lake, our investigations point to a low risk of infection during spring and early summer (March-June) due to no or low shedding of infective *T. szidati* cercariae from the resident snails. When the developing sporocysts in the snails mature and release cercariae in late summer, as seen in August, the risk of infection is high. During our samplings 100 to 200 snails were easily collected within 1 h suggesting a total snail population in the lake of several thousand individuals. As the prevalence was 10% and each snail has a potential to shed up to 77,000 cercariae per day in a small lake with a water depth below 1.5 m and surface area of less than 10,000 m^2^ the concentration of cercariae must be high. Survival of the snail and its shedding capacity are important epidemiological parameters and should be included in future risk assessments. This may further be emphasized by finding that the cercarial life span (for at least 10% of the cercariae) survive 60 h post-shedding at 20 °C and 36 h at 25 °C whereby a steady increase of the pathogen concentration is expected. The infection pressure may be further elevated by the finding that cercariae due to their positive phototaxy concentrate in the surface water when the public seeks the lake during daytime. Therefore, during the summer period any person entering the lake is likely to get into contact with the parasite and is at potential risk of developing symptoms of cercarial dermatitis. It is therefore recommended to establish a monitoring program for *Trichobilharzia* cercariae in the lake. This can be done by weekly collection of snails and performing shedding experiments as described here. It is known that snail size influence shedding capacity [14, 36, 37]. In our study, the experimental snails had a mean shell height of 44.6 (SD 6.3) mm it is worthwhile to monitor snail size and survival for future risk analyses of the lake.

## Conclusion

The present study used the lake Ringen sø as a model to evaluate the risk of *Trichobilharzia* infection (cercarial dermatitis, swimmer’s itch) in a lake frequented by the public. We demonstrated that infection is caused by cercariae of the bird schistosome *T. szidati* released from the pulmonate snail *L. stagnalis*. Important parameters such as the release (shedding), behavior and life span of the infective larvae are light and temperature dependent. Overall assessment of the risk associated with aquatic activities, is low during spring and early summer (March-June) due to no or a low shedding rate of *T. szidati* cercariae. In contrast, in late summer the risk of infection is high all day. Regular sampling of snails associated with shedding procedures throughout the year are recommended. From early summer weekly samplings of snails and determination of their infection status may assist determination of the infection risk for the public.

## Data Availability

The datasets analysed during the current study are available from the corresponding author on request.

## Prior publication

Data have not been published previously.
